# Effects of transmission-blocking vaccines simultaneously targeting pre- and post-fertilization antigens in the rodent malaria parasite *Plasmodium yoelii*

**DOI:** 10.1186/s13071-016-1711-2

**Published:** 2016-08-08

**Authors:** Li Zheng, Wei Pang, Zanmei Qi, Enjie Luo, Liwang Cui, Yaming Cao

**Affiliations:** 1 Department of Immunology, College of Basic Medical Sciences, China Medical University, No.77 Puhe Road, Shenyang North New Area, Shenyang, Liaoning Province People’s Republic of China; 2 Department of Pathogen Biology, College of Basic Medical Sciences, China Medical University, Shenyang, Liaoning 110001 China; 3 Department of Entomology, The Pennsylvania State University, 501 ASI Bldg., University Park, PA 16802 USA

**Keywords:** Malaria, *Plasmodium yoelii*, Transmission-blocking vaccine, DNA vaccine, Pys25, Pys48/45

## Abstract

**Background:**

Transmission-blocking vaccine (TBV) is a promising strategy for interrupting the malaria transmission cycle. Current TBV candidates include both pre- and post-fertilization antigens expressed during sexual development of the malaria parasites.

**Methods:**

We tested whether a TBV design combining two sexual-stage antigens has better transmission-blocking activity. Using the rodent malaria model *Plasmodium yoelii*, we pursued a DNA vaccination strategy with genes encoding the gametocyte antigen Pys48/45 and the major ookinete surface protein Pys25.

**Results:**

Immunization of mice with DNA constructs expression either Pys48/45 or Pys25 elicited strong antibody responses, which specifically recognized a ~45 and ~25 kDa protein from gametocyte and ookinete lysates, respectively. Immune sera from mice immunized with DNA constructs expressing Pys48/45 and Pys25 individually and in combination displayed evident transmission-blocking activity in in vitro ookinete culture and direct mosquito feeding experiments. With both assays, the Pys25 sera had higher transmission-blocking activity than the Pys48/45 sera. Intriguingly, compared with the immunization with the individual DNA vaccines, immunization with both DNA constructs produced lower antibody responses against individual antigens. The resultant immune sera from the composite vaccination had significantly lower transmission-blocking activity than those from Pys25 DNA immunization group, albeit the activity was substantially higher than that from the Pys48 DNA vaccination group.

**Conclusions:**

This result suggested that vaccination with the two DNA constructs did not achieve a synergistic effect, but rather caused interference in inducing antigen-specific antibody responses. This result has important implications for future design of composite vaccines targeting different sexual antigens.

## Background

Malaria is a devastating disease caused by malaria parasites in the genus *Plasmodium*. In nature, human malaria transmission requires *Anopheles* mosquitoes as obligate vectors. According to a recent WHO report, in 2015 it was estimated that there were 214 million cases of malaria globally (range: 149–303 million), leading to 438,000 deaths (range: 236,000–635,000) [1]. Current tools for combating malaria include vector control with insecticides and artemisinin-based combination therapies [2, 3]. The emergence and spread of drug-resistant parasites over the last four decades, especially with the recent detection of resistance against the front-line treatment artemisinins, highlight the necessity for new control strategies. In this regard, the development of a safe and effective antimalarial vaccine is expected to play an important role in integrated malaria control [4, 5].

Vaccine development efforts have focused on candidate antigens present in the pre-erythrocytic, erythrocytic and sexual stages of the parasites. Sexual stages of the malaria parasites are critical for transmission from humans to mosquitoes. During sexual development, male and female gametocytes in the peripheral blood rapidly differentiate into gametes upon uptake by an *Anopheles* vector. Following fertilization of the male and female gametes, zygotes are formed and develop into motile ookinetes. Ookinetes traverse the peritrophic matrix and midgut epithelium, lodge under the basal lamina of the midgut, and develop into oocysts [6]. A transmission-blocking vaccine (TBV) specifically targets the sexual development of the parasite in the mosquito vector and elicits immunity that effectively blocks transmission of the parasite from humans to mosquitoes [7].

To date, a number of TBV candidates have been investigated and only a handful of antigens show clear evidence of transmission-blocking (TB) activity, including P230, P48/45 and P25 and P28. Pfs48/45 is a pre-fertilization antigen and plays an essential role in parasite fertilization. Targeted disruption of the gene affects the male gamete’s capacity to bind to female gametes [8], and antibodies targeting conformational epitopes of Pfs48/45 prevent fertilization [9, 10]. Furthermore, anti-Pfs48/45 antibodies can be found in human sera from endemic areas, and correlate with TB activity [11–14]. Since pre-fertilization antigens are targets of the natural immune responses, immunity based on such antigens will have the added benefit of natural boosting. The two post-fertilization antigens Pfs25 and Pfs28 are lead targets for the development of TBVs, and are secreted onto the surface of ookinetes. Pfs25 plays vital roles in ookinete survival in the midgut and penetration of the gut epithelium [15]. Mouse antiserum against native [16, 17], or heterologously expressed P25 inhibits parasite development in mosquitoes [18, 19]. Currently, phase I human clinical trials using recombinant Pfs25 and Pvs25 have demonstrated the production of antibodies that significantly inhibit transmission of the parasites, further highlighting their potential for TBV development [20, 21]. Recombinant Pvs25 expressed in yeast induces antibodies that block transmission by up to 80 % in terms of mean oocyst intensity, and by 20–30 % in reduction of prevalence of infection [20].

One important problem associated with TBV is that most recombinant candidate antigens such as Pfs230 and Pfs48/45 require proper conformational folding of target epitopes to elicit functional antibodies [22]. DNA vaccine may overcome the need for such requirements associated with conventional protein immunization and has been shown to induce protective immune responses against several pathogens by eliciting both humoral and cellular immune responses [23–28]. DNA vaccines for Pfs25 have been shown to induce effective TB activity in mice and rhesus monkey [29–31]. Another problem is that most TBVs could not induce sterile TB activity to completely block the development of oocysts in mosquitoes. Thus, it is possible that combining two different TBV candidates that target both pre- and post-fertilization antigens may improve TB activity. To test this hypothesis, we used the rodent malaria parasite *Plasmodium yoelii* as a model system and evaluated the immunogenicity and protective efficacy of DNA vaccines for Pys48/45 and Pys25. We show that these DNA vaccines can induce strong antibody response in mice, and the antibodies are functional in inhibiting zygote and ookinete formation in vitro and blocking oocyst formation in mosquitoes. However, simultaneous DNA immunization against both Pys48 and Pys25 did not achieve an additive effect in the induction of functional TB antibodies.

## Methods

### Mice and parasites

Female BALB/c mice aged from 6 to 8 weeks were used for vaccination and infection with the *P. yoelii* lethal strain 17XL (Py17XL). Infections with the Py17XL blood stages were initiated by intraperitoneal injection of 1 × 10^6^ parasitized erythrocytes per mouse. Parasitemia was determined by microscopic examination of Giemsa-stained thin smears from the tail blood. Mortality of infected mice was recorded daily. All experiments were performed in compliance with the regulations of China Medical University Animal Ethics Committee.

### DNA constructs for immunization

Genomic DNA from Py17XL was used for amplification of the *Pys48/45* and *Pys25* gene. The *Pys48/45* open reading frame (ORF) was amplified using primers 5′-AAG CTT ATG CTC TCC TTT TTT GGG-3′ (*Hin*dIII site underlined) and 5′-GAT ATC TTA TAG CCA CAT AAA AAA TAA GGG AAT-3′ (*Eco*RV site underlined). The *Pys25* ORF was PCR amplified with the primers 5′-GGA TCC ATG AAT ACT TAT TAC AGT GT-3′ (*Bam*HI site underlined) and 5′-CTC GAG TTA AAT GAT ATT TGA GAA TAA TAG-3′ (*Xho*I site underlined). The PCR products were cloned into the DNA vaccine vector pcDNA3.1+ and the resultant plasmids were designated as Pys48/45-pcDNA3.1+ and Pys25-pcDNA3.1+, respectively. After verification of the inserts by sequencing, the plasmids were purified using the EndoFree Plasmid Maxi kit (Qiagen, Germany).

### Immunization scheme

For DNA vaccine, plasmid DNA in 100 μl of phosphate-buffered saline (PBS) was administered into the right and left tibialis cranialis muscles of the mouse by intramuscular immunizations. Mice were divided into five groups. Group 1 received 100 μl of PBS as the negative control. Group 2, 3 and 4 received a dose of 50 μg of empty vector pcDNA3.1+, Pys48/45-pcDNA3.1+ and Pys25-pcDNA3.1+, respectively. Group 5 received 50 μg each of Pys48/45-pcDNA3.1+ and Pys25-pcDNA3.1+ in separate sites, which were not mixed in the same syringe. The injections were administered three times at a four-week interval. Four weeks after the first and each of the two subsequent booster immunizations, pooled sera were collected from all the mice for analysis by enzyme-linked immunosorbent assay (ELISA). For each immunization scheme, five BALB/c mice were immunized.

### Preparation of gametocytes and ookinete culture

The *P. yoelii*-infected blood was diluted in PBS to a hematocrit of 10 % and layered onto a 45 % (v/v) Percoll (Pharmacia GE) PBS cushion. After centrifugation at 350× *g* for 20 min at room temperature (RT), the gametocyte-enriched layer was collected at the interface and washed three times with PBS. Part of the purified gametocytes was then diluted 1:10 with RPMI 1640 medium supplemented with 50 μg/ml of hypoxanthine, 25 mM HEPES, 20 % heat-inactivated fetal calf serum, 24 mM NaHCO_3_, 5 U/ml penicillin, and 5 μg/ml streptomycin (pH 8.4) and cultured at 24 °C for 24 h. The purified gametocyte and the cultured ookinete pellets were lysed by 2 % SDS in PBS at RT, then centrifuged at 12,000 rpm for 10 min at 4 °C. The protein extracts were stored at -80 °C for ELISA and Western blotting.

### ELISA

Serum samples obtained from immunized and control mice were tested for antibodies against Pys48 and Pys25 using ELISA as previously described [19]. Briefly, 96-well microtiter plates were pre-coated (100 μl/well) with 10 μg of purified gametocyte or cultured ookinete antigens in bicarbonate buffer at 4 °C overnight. For estimating endpoint titer of each immunized group, sera from all mice in each immunization group were pooled and diluted from 1:200 to 1:25600 in 1 % bovine serum albumin (BSA) in PBS containing 0.05 % Tween 20 (PBS-T). Plates were incubated for 2 h, washed three times with PBS-T, and incubated with horseradish peroxidase (HRP)-conjugated goat anti-mouse IgG (1:5000) for 1 h, followed by six washes with PBS-T. Finally, the optical density values were measured at 492 nm 20 min after the addition of the substrate.

### Western blot analysis

Protein lysates from purified gametocytes or cultured ookinete pellets of Py17XL were separated by electrophoresis in a 10 % SDS-polyacrylamide gel. Proteins were transferred to a 0.45 μm PVDF membrane (Millipore, USA). The membrane was blocked with 5 % skimmed milk in Tris-buffered saline (TBS), and then incubated for 24 h at 4 °C with pooled mouse antisera at 1:100 in TBS containing 0.1 % Tween 20 (TBST). After three washes with TBST, the membrane was incubated for 1 h with HRP-conjugated goat anti-mouse IgG (Proteintech™, USA) diluted 1:10,000 in TBST. After three washes with TBST, the proteins were visualized with ECL Western Blotting Substrate (Thermo Pierce, USA) and detected using the BioImaging System (Tanon, China). The relative molecular masses of proteins were estimated with PageRuler™ Prestained Protein Ladder (10–170 kDa) (Fermentas, USA).

### Indirect immunofluorescence assay (IFA)

Purified gametocyte or cultured ookinetes were spotted onto multi-well slides, air-dried, and fixed with ice-cold acetone for IFA. Slides were first blocked with PBS containing 5 % skimmed milk for 30 min at 37 °C and then incubated with anti-Pys48 or anti-Pys25 mouse sera (1:50) for 1 h at 37 °C. After rinsing with PBS, the slides were incubated with fluorescein isothiocyanate (FITC)-conjugated goat anti-mouse IgG antibodies (Tago, Camarillo, CA) and the nuclear stain 4′,6-diamidino-2-phenylindole (DAPI) for 30 min at 37 °C. After rinsing with PBS, the slides were mounted under a coverslip in bicarbonate-buffered glycerin, and observed under a fluorescence microscope.

### In vitro zygote and ookinete development assay

To examine whether immune sera possessed TB activity, in vitro zygote and ookinete conversion assay was performed [32]. Ten μl of *P. yoelii*-infected blood was taken from mouse tails on day 3 post-infection and mixed with 90 μl of complete ookinete culture medium containing 20 μl of control or immune sera (1:4). The cultures were incubated at 24 °C for 24 h. Parasites were harvested by centrifugation (500× *g*, 5 min) and the pellet re-suspended in 40 μl of PBS. One μl of the suspension was placed on a slide for fluorescent microscopy and blocked with 5 % fat-free milk for 1 h at 37 °C. After rinsing with PBS, the slides were incubated with Pys25 monoclonal antibodies (mAbs) at 1:200 for 1 h, followed by FITC-conjugated goat anti-mouse IgG for 1 h. The total numbers of zygotes and ookinetes formed per microlitre of the aliquots were counted.

### Mosquito feeding experiment

Four weeks after the last immunization, mice from each of the five groups were inoculated with 1 × 10^6^ Py17XL-parasitized erythrocytes/mouse. Three days after infection, three mice from each group were used for mosquito feeding experiments. Four-day-old female *Anopheles stephensi* mosquitoes were starved overnight and then allowed to feed on Py17XL-infected mice (50 mosquitoes/mouse) for 30 min. The engorged mosquitoes were separated and maintained at 24 °C on 1.5 % fructose and 1.5 % sucrose. Nine days after feeding, mosquitoes were dissected to count infected mosquitoes (prevalence of infection) and oocyst density (number of oocysts/infected midgut) by microscopy. Oocyst density was derived from the dissection of 20–30 mosquitoes per mouse.

### Statistical analysis

Statistical analysis of ELISA data and in vitro TB activity of the immune sera was performed by the GraphPad Prism software. For the ELISA data, one-way ANOVA was used to compare all the immunized groups. The formation of *P. yoelii* zygotes and ookinetes was analyzed by the Mann-Whitney U-Test. Considering the over-dispersion nature of the oocyst density and prevalence distribution, these data were analyzed by using a zero-inflated Generalized Linear Mixed-Model statistical model (GLMM) [33]. A value of *P* < 0.05 was considered significant.

## Results

### Antibody responses to DNA vaccines

**Fig. 1:**
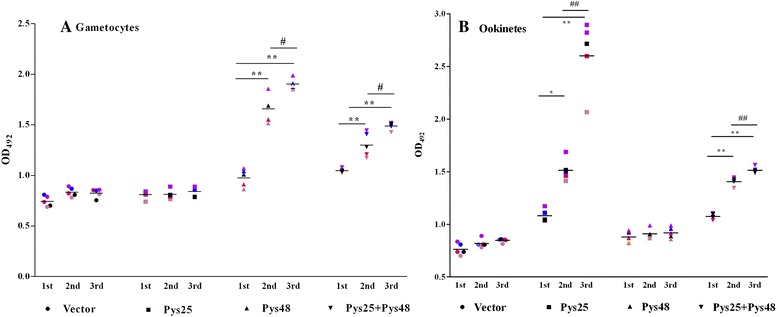
The levels of specific IgGs in serum samples from BALB/c mice immunized with Pys48/45 and Pys25. The coating antigens used were lysate of purified gametocytes (**a**) and of cultured ookinetes (**b**). Results are expressed as geometric means of five in each group. Statistical analysis was performed by one-way ANOVA using the GraphPad Prism software. Asterisks (*) and (**) indicate significance at *P* < 0.05 and *P* < 0.01, respectively, as compared with the first immunization. Octothorps (#) and (##) indicate significance at *P* < 0.05 and *P* < 0.01, respectively, compared between second and third immunization. The data are representative of two separate experiments. Each mouse at the three immunization time points is coded with the same color and symbol

Current TBV designs target pre-fertilization antigens such as P48/45 or post-fertilization antigens such as the major ookinete surface antigen P25. To test whether a combination vaccine targeting both pre- and post-fertilization antigens would perform better, we tested the combination of Pys48/45 and Pys25 using the rodent malaria parasite *P. yoelii* as a model. DNA vaccine constructs were used in order to circumvent difficulties associated with producing correctly folded recombinant proteins of the two Cys-motif proteins Pys48/45 and Pys25 in prokaryotic expression systems. Groups of five mice were immunized with DNA vaccines against either individual antigens (Pys48/45 or Pys25) or combination antigens (both Pys48/45 and Pys25). Antibody responses were measured by ELISA with whole cell lysates of purified gametocytes or cultured ookinetes. Compared with mice in the control group, immunization of mice with the empty pcDNA3.1+ vector did not produce any noticeable antibody response against parasite sexual stage antigens. Consistent with the abundant expression of Pys48/45 in gametocytes and Pys25 in ookinetes, immunization with Pys48/45-pcDNA3.1+ individually and in combination with Pys25-pcDNA3.1+ produced significantly higher antibody titers to the gametocyte lysate than the empty vector, whereas immunization with Pys25-pcDNA3.1+ individually and in combination with Pys48/45-pcDNA3.1+ produced significant higher antibody responses to the ookinete lysate than the empty vector (Fig. 1a, b). The antibody production showed a significant increase in each immunized mouse after the first and second boost (Fig. 1a, b). These results suggested that mice immunized with both DNA vaccine constructs produced specific antibodies against either gametocyte or ookinete antigen(s). Intriguingly, vaccination with both constructs simultaneously resulted in lower antibody titers than those immunized with the two constructs individually (Fig. 1).

### Reactivity of the immune sera with Pys48/45 and Pys25 proteins

**Fig. 2:**
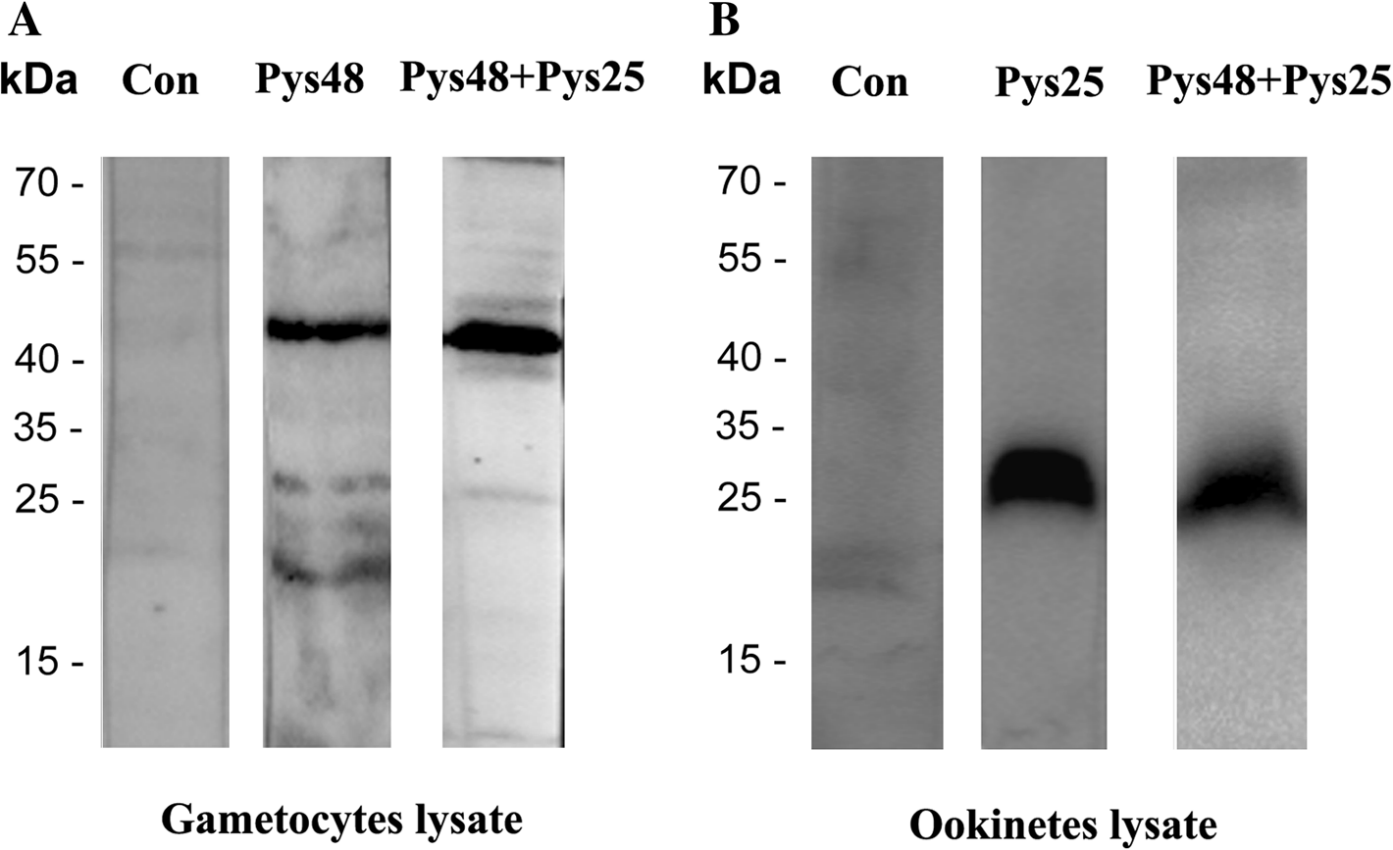
Western blot analysis of Pys48/45 and Pys25 expression in *P. yoelii* gametocytes and ookinetes. Lysates from gametocyte- (**a**) and ookinete-enriched (**b**) preparations were probed with immune sera from the Pys48/45, Pys25 and Pys48/45 + Pys25 DNA immunization groups, respectively

**Fig. 3:**
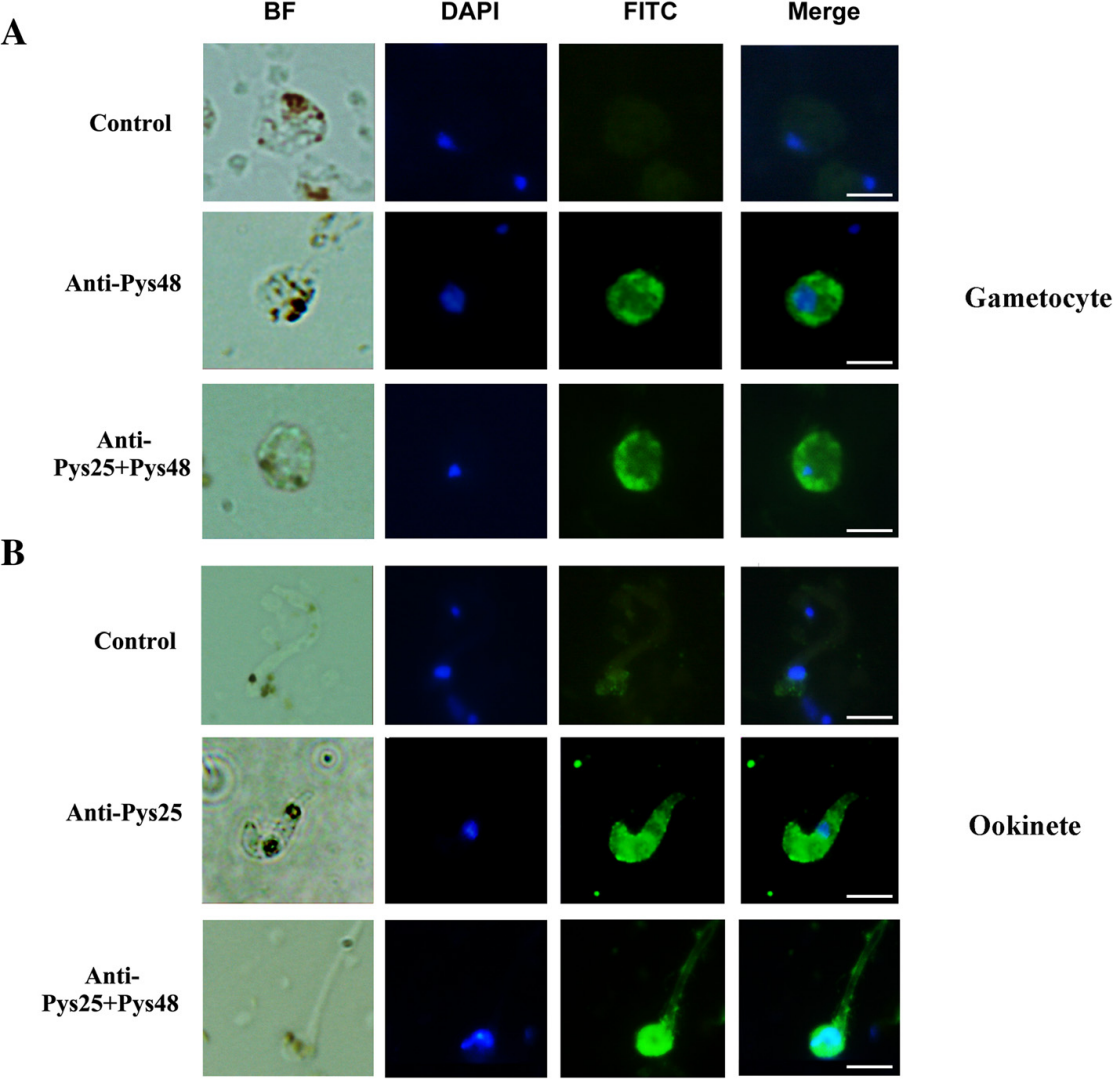
Indirect immunofluorescence assays showing the reactivity of the immune sera with *P. yoelii* sexual stage parasites. IFAs were performed on gametocytes (**a**) and ookinetes (**b**) with immune sera from the Pys48/45, Pys25 and Pys48/45 + Pys25 DNA immunization groups, respectively. Nuclei were counter-stained with DAPI. *Scale-bars*: 5 μm

In order to confirm that the specific antibodies produced against gametocyte and ookinete lysates were indeed specific for Pys48/45 and Pys25, respectively, we performed Western blot and IFA analyses. Western blots showed that the immune sera from Pys48/45-pcDNA3.1+ vaccinated mice detected an approximately 45 kDa protein in the gametocyte lysate, whereas the immune sera from Pys25-pcDNA3.1+ vaccinated mice detected a ~25 kDa protein in the ookinete lysate (Fig. 2), indicating that these antisera specifically recognized Pys48/45 and Pys25, respectively. Sera from combination antigens (both Pys48/45 and Pys25) immunized group detected both the 45 kDa protein and the 25 kDa protein, indicating that the antisera recognized both Pys48/45 and Pys25 (Fig. 2). Although there were minor cross-reacting bands in the Western blot with the gametocyte lysates, the dominant bands were consistent with the predicted molecular weight of the Pys48/45. Furthermore, IFA demonstrated that these immune sera reacted predominantly with the surface of Py17XL gametocytes and ookinetes, respectively (Fig. 3).

### In vitro TB activity of the immune sera

To assess TB activity of the immune sera from the DNA vaccines, in vitro zygote and ookinete inhibition assay was performed using these immune sera at 1:4 dilution. Zygote and ookinete formation was quantified using IFA to detect the major ookinete surface antigen Pys25. Compared with the control sera, sera from the vector control group did not have any inhibitory activity on zygote and ookinete formation (Table 1). In contrast, the three immunization groups all exhibited significantly higher inhibitory activity on zygote formation than the control group (ANOVA: *F*_(3, 12)_ = 194.6, *P* < 0.0001). The numbers of zygotes formed with sera from the three immunization groups were reduced by at least 7-fold compared with the control groups (Table 1). Similarly, incubation with sera from the immunization groups resulted in at least 23-fold reduction in the number of ookinetes (Table 1). The best inhibitory activity was from the Pys25-pcDNA3.1+ vaccination group, where zygote formation was reduced by ~17-fold and ookinete development was completely blocked (ANOVA: *F*_(3, 12)_ = 74.67, *P* < 0.0001). Consistent with the observed lower parasite-specific antibody titers induced with the combination immunization, sera from mice immunized with Pys48/45-pcDNA3.1+ and Pys25-pcDNA3.1+ together did not show higher inhibitory activity on zygote and ookinete formation than sera immunized with these constructs individually (Table 1).

**Table 1:** Inhibitory activity of immune sera from the DNA vaccines on the in vitro formation of *P. yoelii* zygotes and ookinetes

Group	Median no. of parasites/well (range)		% inhibition of ookinetes
	Zygotes	Ookinetes	
Naïve mice	45.0 (39–51)	13.5 (8–17)	
Vector control	42.0 (37–47)	11.5 (9–14)	14.8
Pys25	2.5 (1–4)^a^	0.0 (0–1)^a^	100
Pys48	4.5 (3–6)^a^	0.5 (0–1)^a^	96.3
Pys25 + Pys48	6.0 (4–8)^a^	0.5 (0–1)^a^	96.3

^a^Significant difference compared with the Naïve mice group at *P* < 0.05 by the Mann-Whitney U-Test

### TB activity in mosquito feeding experiments

**Fig. 4:**
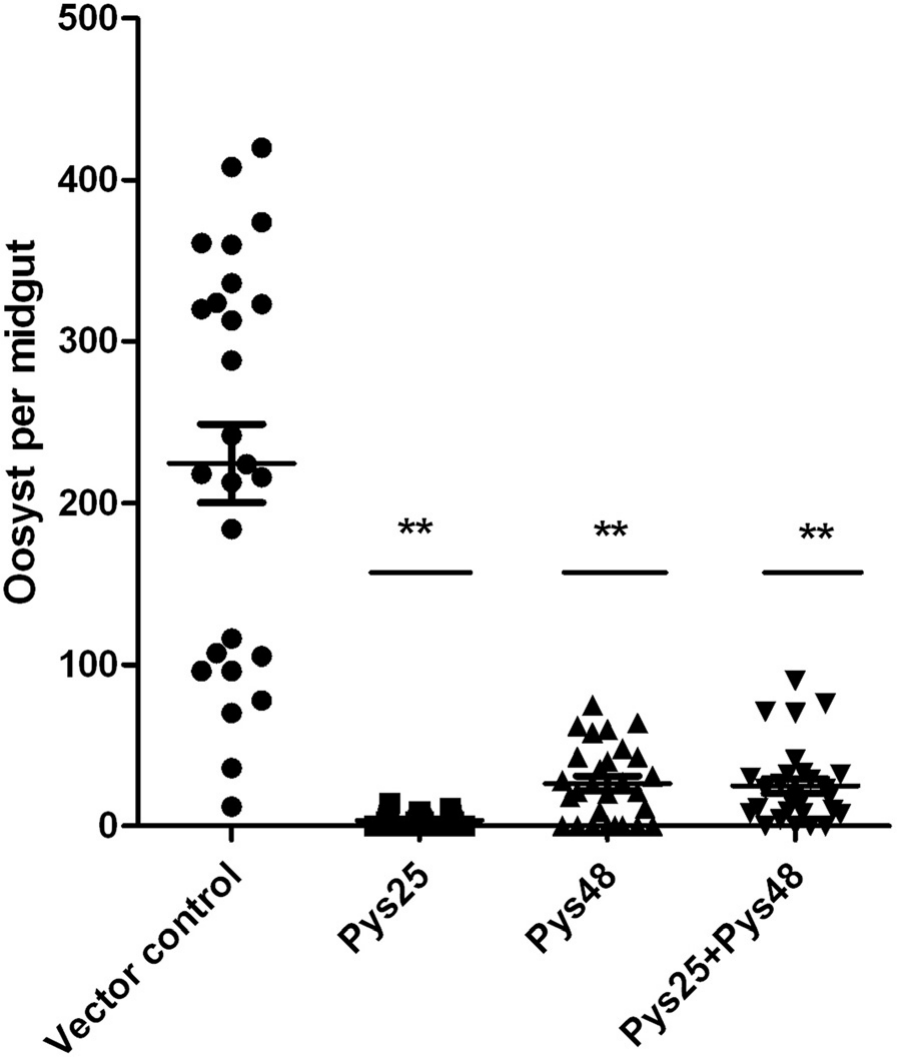
Transmission-blocking effect of immunization with Pys25, Pys48/45 and Pys48/45 + Pys25 plasmids in direct mosquito feeding assay. Data points represent the number of oocysts in individual mosquitoes, horizontal bars indicate the mean number of oocysts per midgut and error bars indicate SEM within individual treatments. Three independent experiments were performed. Asterisk (**) indicates significance at *P* < 0.01 as compared with the Vector control

To compare the TB activity of different immunization schemes, *An. stephensi* mosquitoes were used to feed on mice in the different immunization groups on day 3 after Py17XL infection. Mosquitoes were dissected on day 9 after feeding to determine the mosquito infection rate and oocyst density. In the control group, 100 % mosquitoes were infected with median oocyst density exceeding 200 oocysts per midgut. In comparison, mice receiving the DNA vaccines Pys48/45-pcDNA3.1+ and Pys25-pcDNA3.1+ either individually or in combination significantly reduced the prevalence of infected mosquitoes and oocyst density (*P* < 0.001, Fig. 4). Compared with 100 % infectivity in the control group, immunization with Pys25-pcDNA3.1+, Pys48/45-pcDNA3.1+ and their combination led to 40.7, 28.6, and 14.3 % reduction in the prevalence of infected mosquitoes, respectively. In addition, the average number of oocysts per midgut was reduced to 3, 23.5 and 18.5 oocysts/midgut in these immunization groups, respectively (Table 2). Again, immunization with Pys25-pcDNA3.1+ produced the best TB activity. Further, the combination immunization group, although appeared slightly better than the Pys48/45-pcDNA3.1+ immunization group, showed lower TB activity than Pys25-pcDNA3.1+ single immunization in both the prevalence of infection and oocyst density.

**Table 2:** Transmission-blocking effect anti-Pys25 and Pys48/45 sera in direct mosquito feeding assay

	Vector control	Pys25	Pys48	Pys25 + Pys48
N	26	27	28	28
# not infected	0	11	8	4
Prevalence (%)^a^	100.0	59.3	71.4	85.7
Median	221.0	3.0	23.5	18.5
Mean	224.6	3.6	26.4	24.9
Range	420	14	75	71
Reduction in Oocyst (%)^b^	na	98.6	89.4	91.6
Reduction in Prevalence (%)^c^	na	40.7	28.6	14.3

^a^The prevalence of infection was calculated by number of mosquitoes with oocysts/total mosquitoes dissected in each group

^b^% reduction in oocyst was calculated as (median_control_–median_pcDNA3.1 + plasmid_)/median_control_ × 100

^c^% reduction in prevalence was calculated as (% prevalence_control_–% prevalence_pcDNA3.1 + plasmid_)/  % prevalence_control_ × 100

## Discussion

Due to the complex nature of the malaria parasite’s life cycle, the development of multi-stage vaccines targeting the major stages (pre-erythrocytic, asexual blood and sexual stages) is a more effective vaccination strategy. In addition, mosquito midgut proteins are alternative TBV candidates [34]. Immune responses to individual immunogens in vaccine cocktails have been observed [35, 36], and vaccines targeting more antigens or epitopes may provide better protection than those for single antigens or epitopes in animal models [37–39]. Vaccines targeting antigens of the same stage are expected to have a synergistic effect, however, this may not always be the case. For example, antibodies to Pfs25 and Pfs28 were found to have synergistic TB activity [40], whereas antibodies to the orthologous Pvs25 and Pvs28 did not show obvious synergism [41]. Given that blocking individual stages requires different stage-specific antibody titers, combination of antigens in cocktail vaccines may need to be evaluated on a case-by-case basis [42].

DNA-based vaccines have been shown to generate both cellular and humoral immune responses in diverse animal models. In this study, we evaluated the TB effect of combination DNA vaccines against two sexual-stage antigens in a rodent malaria model. The two antigens tested, P25 and P48/45, are both leading TBV candidates. Since the immunogenicity of a vaccine depends on the formation of natural conformational epitopes, and it is especially important for P48 which contains a unique arrangement of six cysteine-containing domains [43], we expect that DNA vaccine would circumvent the difficulties in obtaining conformationally correct P48 [44] as shown for the Pvs48/45 [45]. Consistent with DNA vaccine results for the Pfs25 [29] as well as the Pvs48/45 [45], DNA vaccines with the Pys25 and Pys48/45 individually and in combination produced evident, specific antibody responses, and the antibodies recognized the respective native proteins in parasites and possessed significant TB activity. In both in vitro ookinete conversion and direct mosquito feeding assays, immunization with the Pys25 plasmid produced much better TB activity than with the Pys48/45 construct, which agrees with P25 being one of the best TBV candidates in numerous experiments [22, 46]. As in a vaccination study with recombinant Pfs25-Pfs230, immune responses are strongly biased towards Pfs25 [47]. This could be due to a difference in the distribution and immunogenicity of the B epitopes, which may be inherent characteristics of individual proteins. More surprisingly, immunization with both DNA constructs produced decreased humoral responses to each of the two antigens as compared with immunization with individual DNA constructs, suggesting of interference between the two DNA vaccine constructs. This phenomenon is consistent with an earlier report showing a similar interference effect of DNA vaccination with Pfs25 and the gametocyte antigen Pfg27, which showed lower TB activity in the combination vaccination group [29]. Alternatively, the reduced antibody responses to individual antigens produced in the mixed vaccination scheme could be a dosage effect, as the doubled amount of DNA used for combination immunization may interfere with antigen presentation.

## Conclusion

The data described here indicate that both Pys25 and Pys48/45 DNA vaccines showed remarkable immunogenicity and induced functional TB activity. Though both antigens are considered leading TBV candidates targeting pre- and post-fertilization antigens respectively, their combination in immunization produced an interfering effect in eliciting immune responses to either protein. It remains to be tested whether this is a phenomenon restricted to DNA vaccine only. Nonetheless, these results could have important implications in the design of future multicomponent DNA vaccines.

## Abbreviations

BSA, bovine serum albumin; DAPI, 4′,6-diamidino-2-phenylindole; ELISA, enzyme-linked immunosorbent assay; FITC, fluorescein isothiocyanate; GLMM, Generalized Linear Mixed-Model statistical model; HRP, horseradish peroxidase; IFA, Indirect immunofluorescence assay; ORF, open reading frame; PBS, phosphate-buffered saline; Py17XL, *P. yoelii* lethal strain 17XL; RT, room temperature; TB, Transmission-blocking; TBS, Tris-buffered saline; TBV, Transmission-blocking vaccine
